# Guidelines for experimental design and statistical analyses in animal studies submitted for publication in the Asian-Australasian Journal of Animal Sciences

**DOI:** 10.5713/ajas.18.0468

**Published:** 2018-07-26

**Authors:** Seongwon Seo, Seoyoung Jeon, Jong K. Ha

**Affiliations:** 1 Division of Animal and Dairy Science, Chungnam National University, Daejeon 34134, Korea; 2 Asian-Australasian Journal of Animal Sciences, Seoul 08776, Korea; 3 Department of Agricultural Biotechnology, College of Agriculture and Life Science, Seoul National University, Seoul 08826, Korea

**Keywords:** Statistical Analysis, Asian-Australasian Journal of Animal Sciences, Guidelines

## Abstract

Animal experiments are essential to the study of animal nutrition. Because of the large variations among individual animals and ethical and economic constraints, experimental designs and statistical analyses are particularly important in animal experiments. To increase the scientific validity of the results and maximize the knowledge gained from animal experiments, each experiment should be appropriately designed, and the observations need to be correctly analyzed and transparently reported. There are many experimental designs and statistical methods. This editorial does not aim to review and present particular experimental designs and statistical methods. Instead, we discuss some essential elements when designing an animal experiment and conducting statistical analyses in animal nutritional studies and provide guidelines for submitting a manuscript to the Asian-Australasian Journal of Animal Sciences for consideration for publication.

## INTRODUCTION

For scientific, ethical, and economic reasons, experiments involving animals should be appropriately designed, correctly analyzed, and transparently reported. This increases the scientific validity of the results and maximizes the knowledge gained from each experiment. Nonetheless, biologists, on average, feel uncomfortable with mathematics and statistics, and they often design experiments and analyze data in inappropriate ways [1]. Therefore, in some fields of research where animal experiments are essential, the editorial board regularly reviews the statistical methodologies reported in the papers and presents their suitability [2–5]. Some fields of research have set up consortia and provide guidelines for animal experiments [6,7], and some scientific journals have guidelines for their authors to follow for publication [8,9]. For example, in the animal science field, the Journal of Dairy Science provides detailed guidance on statistical methodology in the instructions to authors [10]. Animal Feed Science and Technology has published two editorials that discuss proper experimental design and statistical analyses to guide authors who are submitting manuscripts to the journal [11,12].

The Asian-Australasian Journal of Animal Sciences (AJAS) published the first issue in January 1988, and its contribution and influence to the animal science fields have continuously expanded over the past three decades. In particular, a total of 102 nutritional studies were published in AJAS in 2017, which included 84 *in vivo* trials. In these studies, statistical methods are essential, and authors should strive to employ an appropriate experimental design and statistical analyses to provide the reader with scientifically relevant and valid knowledge.

This editorial will discuss some of the principles of experimental design and statistical analysis and provide guidelines when submitting nutritional studies to AJAS for consideration for publication.

## EXPERIMENTAL DESIGN

Authors must provide details regarding the experimental design in a manuscript such that reviewers and readers have sufficient information about how the study was conducted and can evaluate the quality of the experimental design. Details include animal characteristics (e.g., species, breed, gender, weight), number of treatments, number of experimental and sampling units, arrangement of treatments (e.g., factorial, change-over), and consideration for known variation (e.g., blocking, covariates). Only properly designed experiments can yield valid and reliable results that lead to correct and appropriate interpretations and conclusions in the study.

### The experimental unit and the number of replicates

Treatments, the set of circumstances created for an experiment based on research hypotheses, are the effects to be measured and compared in the experiment [13]. The treatment is applied to the experimental unit, and one experimental unit corresponds to a single replication; Kuehl [14] defines the experimental unit as “the physical entity” or subject exposed to the treatment independently of other units. The number of replicates (i.e., sample size) is the number of experimental units per each treatment. Defining the experimental unit correctly is crucial for proper experimental design and statistical analysis. However, correctly defining the experiment unit is sometimes not easy. This is especially true in the cases where a group of animals are fed together in a pen, there is debate as to the most appropriate experimental unit between statisticians and biologists [11].

Like most other biostatisticians [14,15], editors of AJAS have a more conservative view regarding the determination of the experimental unit. For many nutritional studies, the purpose of the experiment is to infer population means. For example, in a feeding trial in which different dietary treatments are applied to different groups of animals, the ultimate goal of the experiment is not to observe the treatment effect within the experimental animals but to investigate its effect on independent animals in the real world. The role of replication is to provide measures of how much the results are reliable and reproducible, and thus replicates are to be independent observations and experimental units must be independent of each other. If a treatment is applied to a group of animals in a single pen, the individual animals are not independent; thus, the pen is considered the experimental unit even when measurements are made individually. The treatment effect is confounded by the effect of the pen in this case, and it is obvious that the pen should be the experimental unit because it is unknown whether the results of the experiment were caused by the treatment of the pen. On the other hand, if treatments are randomly assigned to individual animals within a group of animals in a pen, the individual animal can be considered the experimental unit even though they are in the same pen.

A sufficient number of replicates are needed to obtain a reliable outcome from an experiment. Because the number of replicates is related with the power of a test, more experimental replicates can provide greater statistical power to detect a desired difference among treatments. The cost of replicates, however, is high in animal experiments, and the smallest number of replicates is preferred, as long as it is sufficient to detect a difference. For this purpose, power tests are performed prior to initiating an experiment to determine the required sample size based on expected variation in means and the size of the difference between means that needs to be detected.

Power tests are also useful for supporting the validity of an experiment when no significant difference is observed between the treatment means. It is not uncommon to fail to detect a significant difference between treatments, and in this case, one can argue that significance was not observed simply because the sample size was small. The result from the power test can provide supportive evidence that the reason for the failure to detect a difference between treatments was not because the sample size was small, rather the difference between the treatment means was not great enough to be considered significant.

Therefore, AJAS encourages authors to provide the results of power tests. The results of power tests can be used to justify that the experiment was appropriately designed.

### Consideration for known variations

To properly test for treatment effects, factors other than the main treatment that may affect the response of the animals should be minimized or at least accounted for. In this regard, the use of a block or covariate is recommended.

Blocking is a practice wherein the experimental units are placed into groups of similar units based on one or more factors that are known or expected to affect the responses to be measured and tested. Physical and physiological characteristics, such as sex, litter, and initial body weight, are commonly used for blocking in the animal science field. Blocking controls the variability of experimental units and reduces experimental error.

Covariates are variables that are known or expected to be related to the response variables of interest. The primary difference between blocks and covariates is that covariates are continuous variables, whereas blocks are categorical variables. For example, animals can be grouped or blocked as high, medium, and low groups according to their body weight. Conversely, individual body weight can be used as a covariate to reduce the estimates of experimental error in the statistical model. Blocking is applied at the experimental design stage, whereas the use of covariates is applied when conducting statistical analysis.

The use of a block and covariate is a sound and logical way to account for known errors and reduce unexplained errors. The AJAS editorial board thus encourages authors to use blocks and covariates if there are known or expected variables that could have a significant effect on the response to be tested for in the experimental treatments.

When a limited number of animals are available or when individual animal variation is to be removed, crossover (i.e., changeover) designs are often used in animal nutritional studies. In this case, it can be an issue if a carryover effect from a treatment given in a previous period influences the response in the following treatment. It should be noted that crossover designs should be avoided when significant carryover effects are expected [16]. Even if a significant carryover effect is not expected, the potential for a carryover effect should not be ignored in crossover designs. A sufficient rest or wash-out period between two treatment periods is one of the practical ways to minimize carryover effects. More importantly, the order of treatments for each animal should be balanced to avoid confounding of treatment and period effects and to minimize the influence of carryover effects. In a balanced crossover design, each treatment follows each of the other treatments an equal number of times, and each treatment is assigned once for each animal and the same number of times in each period. When a carryover effect is suspected, its significance also needs to be tested by statistical analysis. The AJAS editorial board recommends authors describe the procedure used to minimize possible carryover effects and show that carryover effects are not significant in their study when using a crossover design.

### Randomization

Randomization is an essential procedure to ensure the reliability of the experiment and the validity of the statistical analysis. The purpose of an experiment is to make inferences about the population mean and variance, and the statistical analysis assumes the observations are from a random sample from a normally distributed population. This assumption can be valid only through randomization. In animal nutritional studies, two randomization processes are required: random sampling of experimental units and random allocation of treatments to experimental animals.

Theoretically, experimental animals represent the animal population of interest; thus, they need to be randomly selected from the population. However, this is usually not feasible, if not impossible, in the real world and whether experimental animals can be considered a random sample is questionable. Nevertheless, whenever possible, randomization must be practiced in selecting experimental animals to eliminate biases and to obtain valid estimates of experimental error variance. For example, when a deep analysis is performed on selected animals (e.g., blood analysis for selected animals from a group of animals in each treatment), random selection should be conducted.

Random allocation of treatments to experimental units is the most important and critical step to justify and establish the validity of statistical inferences for the parameters of the population and tests of hypothesis. The experimental errors are assumed to be independently and normally distributed. Estimation of parameters and statistical inferences can be possible if and only if this assumption is valid. Random assignment of treatments to experimental animals is the only method that guarantees the independence of observations and permits us to proceed with the analysis as if the observations are independent and normally distributed. The authors are required to describe the randomization procedure used for their animal trials.

## STATISTICAL ANALYSIS

Statistical analysis is conducted to test the hypotheses and significance of tests in a study. There are many methods for conducting statistical analysis and various methods yield different results and conclusions. Proper statistical methods should be applied when conducting an experiment, and details of statistical methods should be provided in the statistical methods section of a manuscript to allow reviewers and readers to assess the quality of statistical methods used in the study.

### Statistical models

When submitting a manuscript for publication in AJAS, authors should clearly define their statistical models used for the statistical analysis. Statistical models are usually expressed as linear models with the overall mean of the response variable, fixed or random variables that are known to influence the response variable, and unexplained experimental random error. The statistical model should be consistent with the experimental design and be appropriate to analyze the observations from the experiment. A clear description of the statistical model as an equation, as well as in words, is useful to understand the analytical procedure and the meaning of statistical implications and to evaluate the correctness and relevance of the statistical methods used in the study. Thus, the statistical model is often used as a criterion for the recommendation of manuscript rejection by reviewers and editors [11].

### Statistical methods

Various statistical methods are available, and the choice of method depends on the data type of observations, research questions to answer, and the statistical model.

If observations of the response variables are binary (i.e., yes or no) or categorical, the logistic model or other categorical analysis needs to be used. Sometimes research questions are not about means but seek to understand the quantitative relationship between response variables or between the response variable and treatment (e.g., dose-response analysis). The linear or non-linear regression analysis is the method to be used in this case.

When the response variable of interest is a continuous variable and the research question is about means or an interval of the value, either parametric or non-parametric statistical methods can be applied. The most famous parametric statistical methods are the t-test and analysis of variance (ANOVA). A t-test is used for comparing two samples or treatments, whereas the ANOVA is used when there are more than two treatments. Different methods can be used within a t-test and an ANOVA. For example, if two samples are paired (e.g., blood samples collected before and after treatment in the same animal), a paired t-test is most appropriate. Additionally, because different levels of complexity can exist in statistical models (e.g., the existence of both fixed and random effects and their interactions, repeated measures over time), the most appropriate method may vary by the statistical models when conducting an ANOVA. Parametric methods assume that the observations are independent and normally distributed around their mean. This assumption is generally true in animal nutritional studies as long as randomization is practiced. However, it is always a good practice to test this assumption, especially if variables are expected not to follow it. For example, particle size normally has a log-normal distribution [17], and thus statistical tests need to be performed on transformed values.

If the observations are not normally distributed or the sample size is not large enough, non-parametric analyses (e.g., Mann-Whitney U test instead of a t-test and Kruskal-Wallis H test instead of a one-way ANOVA) would be the methods of choice. Non-parametric methods do not assume a normal distribution of experimental errors and more powerful to detect differences among treatments than parametric methods (e.g., t-test and ANOVA). Because non-parametric methods have more statistical power, they can exaggerate the significance of the difference between treatments. A parametric method is thus preferred when it is applicable.

### Comparing the means of interest

When an ANOVA reveals that the probability that treatment means are all equal is sufficiently small enough to conclude that at least one of the treatment means is different from the others, we may ask further questions, such as which ones are different from each other? Before conducting further analyses, two things are to be considered.

First, we need to determine how small is sufficiently small. This is called the level of significance, and it is normally assumed that the probability of less than 5% (i.e., p<0.05) is statistically significant in animal nutritional studies. The level of significance is also called type I error or α, which is the probability of rejecting a null hypothesis when it is true. If α = 0.05, the test can mistakenly find treatment effects in a maximum of one out of 20 trials. When the p-value obtained using an ANOVA test is less than the level of significance, the results may be meaningful and need to be discussed; thus, comparing the means becomes interesting. If the obtained p-value is larger than the predetermined level of significance, we need to conclude that the null hypothesis is plausible, and we do not have enough evidence to reject the null hypothesis and accept the alternative hypothesis. It should be pointed out that we must not accept the null hypothesis. It is logically impossible to test whether the null hypothesis is true and to prove all the means are the same. We cannot ensure that the null hypothesis would remain plausible if the number of replicates was larger. The authors are thus required to state the level of statistical significance in the statistical analysis section.

Next, we need to determine which techniques are most appropriate for the post hoc analysis on the basis that there is a significant difference among the treatments using an ANOVA. One of the most intuitive and simplest methods to compare the means of interest is linear contrasts. If the number of treatments is t, then a set of t – 1 orthogonal contrasts can be tested. Sets of orthogonal contrasts are not unique for a given experiment; there may be many such sets. Finding an appropriate set of orthogonal contrasts lies in the structure of the treatments. For example, suppose there is an experiment of testing two feed additives as alternatives to antibiotics, and it has four treatments: without feed additives (CONT), antibiotics (ANTI), feed additive A (ADTA), and feed additive B (ADTB). A set of 3 (4 – 1) orthogonal contrasts that can be made, and logical and obvious contrasts are i) CONT vs the others, ii) ANTI vs (ADTA and ADTB), and iii) ADTA vs ADTB.

In addition to linear contrasts, there are many methods available for multiple comparisons of means; the most widely used methods include Dunnett’s test [18], Tukey’s test [19], Scheffe’s test [20], the least significant difference (LSD) [21], and Duncan’s multiple range test [22]. Among these, Duncan’s test is the most popular method in the animal nutritional studies. Approximately 37% of animal nutrition papers that conducted pair-wise comparisons in 2017 in AJAS used Duncan’s test. The second most used tests were the LSD and Tukey’s test; each accounted for 14% of multiple comparison tests.

The AJAS editorial board does not take a position on which test is more desirable under certain circumstances and leaves the decision to authors as long as the test can properly test logical questions according to the experimental design. For example, for a dose-response experiment with increasing inclusion levels, testing the significance of differences between particular means is inappropriate. Instead, linear and curve-linear regression for testing the dose-response relationship would be a better choice. A pairwise comparison procedure is appropriate to use when there is no structure among a series of treatments.

### Statistical software packages and statistical procedures

There are several software packages available for statistical analysis. Even using the data analysis add-in of Microsoft Excel allows for the t-test, analysis for correlation, linear regression analysis, and one-way ANOVA to be performed. More complicated statistical models, however, require software with statistical packages, which include the statistical analysis system (SAS), general statistics (GENSTAT), statistical program for the social sciences (SPSS), Minitab, and R. The most commonly used statistical software in animal nutritional studies is SAS. Fifty-five percent of animal nutrition papers published in 2017 in AJAS used SAS. The second most popular statistical software was SPSS (27.5%), and more than 83% of the papers used one of them. Like other journals, AJAS takes no position on which of these statistical software packages is more desirable in any particular circumstances and leaves that decision to authors. However, it is required for authors to report which software is used for the statistical analysis.

Even within each statistical software package, there are different procedures that can be used for analyzing data. For example, when conducting an ANOVA in SAS, any procedures that can solve a general linear model, such as the ANOVA, GLM, and MIXED procedures, can be used. However, each procedure may have different features and work better for a specific circumstance. For example, in SAS, compared with the GLM procedure (PROC GLM) which is designed to analyze a general linear model with fixed effects, the MIXED procedure can better handle statistical models having random effects. For the analysis of binary or categorical variables with fixed effects, the GENMOD procedure that uses a generalized linear model should be used instead of PROC GLM. A more recent procedure, PROC GLIMMIX, can analyze statistical models with fixed and random effects for both categorical and continuous variables. AJAS does not take a position on which procedures are more desirable under certain circumstances and leaves the decision to authors as long as the procedure can properly handle the data type. However, when the observations are repeatedly measured or random effects are included in the statistical model, PROC MIXED or PROC GLIMMIX in SAS or similar procedures in other statistical packages are preferred.

## REPORTING

Reporting all relevant information is important in scientific papers to increase the transparency and validity of the results and provide information for confidence and limitations of scientific knowledge gained from experiments. Not only the probability value (p-value) but also error measures (e.g., standard error of means [SEM]) should be reported in tables. Likewise, error measures should be present as error bars in figures. Error measures can be expressed in several ways: standard deviation (SD), SEM, and the standard error of the difference (SED). AJAS recommends the presentation of the pooled SEM because the objective of animal nutritional studies is usually to provide inferences about the population. If the sample sizes are different among treatments, the sample sizes are to be reported, as well as pooled SEM. However, the use of SD is also allowed when it is used for descriptive statistics.

When there are outliers or missing data, they need to be clearly reported in the Materials and Methods section or the Results section of the manuscript where it is more appropriate. In particular, the methods and their rationale for identifying outliers should be provided, and the results from the statistical analysis of the data with and without outliers should be compared and discussed in the manuscript.

## SUMMARY OF RECOMMENDATIONS

The AJAS editorial board takes no position on which experimental designs and statistical methods are more desirable in certain circumstances and leaves that decision to the authors. Nevertheless, a summary of the recommendations of the AJAS editorial board is as follows:

1. Provide details of experimental design and statistical methods in the Materials and Methods section.
2. Define the experimental unit and report the number of replicates. Replicates are to be independent observations and experimental units must be independent of each other.
3. Conduct power tests and provide their results to justify the experiment was appropriately designed.
4. Use blocks or covariates whenever applicable to reduce unexplained experimental errors.
5. Describe the procedure used to minimize possible carryover effects and to show carryover effects are not significant when using a crossover design.
6. Ensure the implementation of randomization when sampling experimental units and allocating treatments to experimental units.
7. Describe the statistical models used for the statistical analysis as equations, as well as in words.
8. Use appropriate statistical methods depending on the data type of observations, research questions to be answered, and the statistical model.
9. Test if the observations are normally distributed around their mean. If not or the sample size is not large enough, use non-parametric analyses instead; otherwise, use parametric methods.
10. State the level of statistical significance in the statistical analysis section.
11. Conduct post hoc analysis on the basis that there is a significant difference among the treatments and use appropriate methods according to the structure of the treatments.
12. Perform pair-wise comparisons (e.g., Duncan’s multiple range test) only when there is no structure among a series of treatments.
13. Report which software and procedures are used for the statistical analysis.
14. Use appropriate statistical methods and procedures if observations are repeatedly measured or random effects are expected.
15. Present both probability value (p-value) and pooled SEM as error measures. The standard deviation can only be used for descriptive statistics.
16. Report outliers or missing data in the Materials and Methods section or the Results section where it is more appropriate.
